# Authority or Autonomy? Exploring Interactions between Central and Peer Punishments in Risk-Resistant Scenarios

**DOI:** 10.3390/e24091289

**Published:** 2022-09-13

**Authors:** Jun Qian, Xiao Sun, Tongda Zhang, Yueting Chai

**Affiliations:** 1 National Engineering Laboratory for E-Commerce Technologies, Department of Automation, Tsinghua University, Beijing 100084, China; 2 Department of Mechanical and Energy Engineering, Southern University of Science and Technology, Shenzhen 518055, China

**Keywords:** central punishment, peer punishment, risk-resistant model, collaboration, evolutionary game theory

## Abstract

Game theory provides a powerful means to study human cooperation and better understand cooperation-facilitating mechanisms in general. In classical game-theoretic models, an increase in group cooperation constantly increases people’s gains, implying that individual gains are a continuously varying function of the cooperation rate. However, this is inconsistent with the increasing number of risk-resistant scenarios in reality. A risk-resistant scenario means once a group does not successfully resist the risk, all individuals lose their resources, such as a community coping with COVID-19 and a village resisting a flood. In other words, individuals’ gains are segmented about the collaboration rate. This paper builds a risk-resistant model to explore whether punishment still promotes collaboration when people resist risk. The results show that central and peer punishments can both encourage collaboration but with different characteristics under different risk-resistant scenarios. Specifically, central punishment constrains the collaboration motivated by peer punishment regardless of risk, while peer punishment limits the collaboration induced by central punishment only when the risk is high. Our findings provide insights into the balance between peer punishment from public autonomy and central punishment from central governance, and the proposed model paves the way for the development of richer risk-resistant models.

## 1. Introduction

Cooperation exists widely in human groups, such as group presentation, economic development [1,2], environmental protection [3,4,5], and healthcare [6,7,8]. Cooperation usually means a win–win situation for most participants, but the existence of free-riding often leads to social dilemmas that ultimately result in the loss of individual and group interests [9,10,11]. Specifically, cooperation requires individuals to contribute their efforts or resources to the group before the rewarding phase, and people who do not contribute or contribute less to the group enjoy the same benefits from group cooperation as those who contribute more. As a result, these people receive more benefits by contributing less and receiving the same. The behavior of these people is known as free-riding behavior. In the long run, free-riding behavior indicates a conflict between individual self-interest and group interest. The pursuit of individual self-interest by too many group members will lead to the failure of group cooperation and eventually make both the individual and the group lose. Therefore, how to solve such social dilemmas to promote cooperation is of great importance to the functioning of society and has received a lot of attention from various scholars [12,13,14,15]. Behavioral economics has proposed various game models [16,17,18] that vividly demonstrate the conflict between the group cooperation and the individual free-riding behavior, such as the dictator game [19,20,21], public goods game [22,23,24,25], prisoner’s dilemma game [20,26,27], etc. In addition, various measures have been proposed to promote cooperation and find a way out of the social dilemma [13,22,28,29,30,31,32,33,34,35].

Many researchers have noted punishment for its cooperation boosting effects [22,35,36,37]. In a nutshell, punishment can reduce the gain of individuals in a group who hold a certain strategy. By changing the relative gains among individuals with different strategies, punishment can affect the attractiveness of different strategies and further limit the spread of free-riding behavior if performed properly [25,38,39,40]. Additionally, punishment does not require external incentives or group restructuring and is highly operational in human societies. Therefore, it is a long-standing hotspot for cooperation researchers to solve different social dilemmas. In terms of who initiated the action, punishment can be enforced either by a central agency [28,41,42,43] or individuals [38,44,45]. One salient form of punishment we can observe is usually from sanctioning institutions, such as police stations, tax offices, and courts. Regardless of the existence of formal sanctioning institutions, punishment could also be imposed decentralized and informally through peers [9,38]. Thus, punishment is divided into two basic types according to who imposes it: central punishment and peer punishment. From the perspective of governance sources, central punishment is an instrument for authority imposed on individuals according to some rules of punishment. In contrast, peer punishment is a means of public autonomy, which refers to an individual paying the price to make the person lose benefits.

Many studies have shown that peer punishment can inhibit free-riding behaviors [22,25,46]. Group members punish free-riding individuals severely so that the benefits received by free-riders may be lower than cooperators. When the advantage of the cooperation strategy outweighs that of free-riding, as rational economic agents seeking to maximize personal gains, free-riding individuals would abandon their approaches in favor of the cooperation strategy. Thus, group cooperation is usually promoted afterward, with everyone betters off higher profits. In this context, a fairly good order of autonomy emerged based on peer punishment. However, the existence of antisocial punishment often handicaps the aforementioned effectiveness of peer punishment because it punishes high contributors [23,47]. Central punishment, as a concrete manifestation of authority, is used in social governance to deal with some free-riding phenomena in real life (e.g., fare or tax evasion). Although central punishment circumvents the adverse effects of antisocial punishment on group cooperation, its sustainability requires group members to pay costs [27]. The continuous fee-paying requirement means that even if no free-riding exists, group members will trade some of their benefits for the existence of central governance on an ongoing basis, which might be considered a waste of resources. In general, peer punishment and central punishment both have their advantages and disadvantages.

To study the cooperation behaviors, most researchers conduct two primary types of games: one is the two-person game between a pair of individuals in a group, such as the prisoner’s dilemma game, the eagle-dove game, etc.; the other one is known as the public goods game played by a group of people. Both types of games have in common that the group gains revenue by completing the project, while in reality, it is increasingly common for groups to bear possible losses due to external risks [31], such as atmospheric pollution [48], overuse of resources [49], and coronavirus pandemics [50,51,52]. In contrast to the traditional game model, everyone is involved in the risk either actively or passively in a risk-resistant scenario. In other words, the impact of risk is global. Everyone suffers when the group fails to defend itself against the threat. From a theoretical point of view, the game’s result is either successful or unsuccessful, then one individual’s payoff becomes a segmented function, which may affect the choice of strategy and the evolution of the group.

In summary, although risk resistance is a very common cooperative behavior in human societies, and punishment is an effective means for humans to promote cooperation, current research on human cooperative behavior has not fully explored the population performance and behavioral patterns in this scenario. The main focus of this paper is on whether punishment is still effective in promoting group collaboration in a risk-resistant scenario. Further, given that central punishment and peer punishment are concrete expressions of authority and autonomy, respectively, how do these two types of punishment affect each other, and how do they differ and interact with each other in promoting group collaboration?

In this article, we propose a risk-resistant game model to explore whether punishment can promote group collaboration in a risk-resistant scenario. Our simulation results show that both central punishment and peer punishment can effectively encourage group collaboration: the stronger the punishment, the better the collaboration. Interestingly, central punishment and peer punishment profoundly influence each other but in a different way. Central punishment constrains peer punishment regardless of risk, while peer punishment limits central punishment only when the risk is large. We also analyze the benefit–cost ratio of the two punishments to understand their interaction. In addition, we investigate resource waste brought by the punishment while promoting group collaboration. Surprisingly, the waste is minute compared to its collaboration promotion influence. Considering the enhancement of collaboration and the amount of wasted resources, we find that in low-risk situations, peer punishment needs to be used primarily but with caution. In high-risk situations, the two punishments need to be balanced, and the central punishment can be slightly more. Our study highlights the significance of risk-resistant scenarios in the study of cooperation and sheds light on the joint use approach of citizen autonomy and central governance in public governance issues.

## 2. Materials and Methods

### Model and Assumptions

The risk-resistant model in this paper makes the following basic assumptions:

- Individuals are rational and their behavior is motivated by maximizing their own gains.
- Individuals choose their own strategies from the strategy space and have the ability to learn strategies from other individuals.
- Individuals as a whole are exposed to risky scenarios, which means that all individuals lose a portion of their gains if the risk is not successfully resisted.

**Figure 1:**
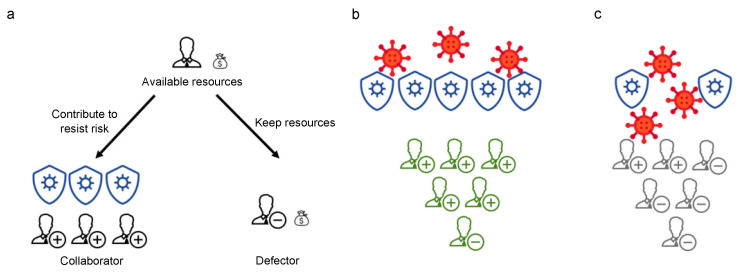
Risk-resistant scenario. Combining the previous research [31] and the model assumptions of this paper, we create the risk-resistant scenario. When group members are at risk, they have two options, as in (**a**) One becomes a collaborator (those marked with a plus sign) and contributes resources to the group. The other become defectors (those with a minus mark) who keep all resources without any contribution. The resources contributed by all collaborators are pooled against the risk. The success of the group in resisting risk depends on their total contributions. When their contribution is above a threshold, as in (**b**), the risk (the red viruses) cannot harm the group members. However, as shown in (**c**), when there are too many defectors and their contribution is below a threshold, the group fails, and everyone loses all resources.

Specifically, we build a risk-resistant model where individuals work together to defend against external risks as shown in Figure 1. Each individual has two choices: to contribute to counteract the risk (collaborator) or not to contribute and rely on others’ contributions to offset the risk (defector). The whole group will face the consequences of the external hazard together, meaning that if they success in counteracting the risk, no one loses any benefit, and everyone loses all their benefits if they fail to do so. We set up both central punishment and peer punishment to punish free-riding behaviors. Specifically, central punishment is enforced on free-riders only if enough funds from group members are able to set up a central enforcement institution; peer punishment, on the other hand, is initiated when an individual is willing to pay a cost to punish free-riders so that free-riders lose more resources than the cost.

Depending on the contribution and the punishment, people in our risk-resistant model are divided into four different types according to their specific strategies:

- Collaborator who does not enforce punishment (C): contributes resources to the group.
- Collaborator who supports central punishment (CP): contributes funds to establish a central enforcement institution and contributes resources to the group.
- Collaborator who enforces peer punishment (PP): pays the cost to punish free-riders and contributes resources to the group.
- Defector who does not enforce punishment (D): does not contribute resources.

It should be noted that there are two other types of people with the other two strategies: the defector who supports central punishment (CD) and the defector who performs peer punishment (PD). However, defectors are free-riders in the group and are also the punishment targets, so these strategies are paradoxical if all group members are rational. Our model ignores the individuals of these two strategies in the following discussion.

In the above risk-resistant model simulation, the group consists of $N=\left(a\cdot n\right)$ individuals. We divide them into *a* groups of *n* people each to play the multi-rounds game. Initially, each individual is randomly assigned a strategy from C, CP, PP, and D. In each round, individuals are first given *m* units of resources and are randomly divided into subgroups of size *n* to play the game within their own subgroup. According to their strategy, everyone decides whether to contribute resources to defend against the risk and pay the corresponding cost (*u*). Then, the process of punishment begins based on individuals’ contributions. Each CP individual invests $w_{1}$ cost in supporting the establishment of a central institution. If sum $\sum w_{1}\geq w$, where *w* is the amount of resources required to establish a central institution, central punishment will be executed toward each D type individual and cause each D individual to lose $t_{1}$ resources; if sum $\sum w_{1}<w$ then no central punishment is executed. For peer punishment, a PP-type individual performs punishment on a D individual at the cost of $w_{2}$ let the D individual loses $t_{2}$ resources ($w_{2}\leq t_{2}$). After the punishment process, the subgroup starts to counter the risk. If the total resources contributed by members ∑u≥U where *U* is the cost needed to resist the risk, the group resists successfully, and everyone keeps their resources. If ∑u<U, the group fails, and everyone loses all resources as a result.

The strategy learning process happens at the end of each round of the game. During the learning process, individuals adjust their strategy according to resource distribution within the group. To be more concrete, individuals tend to imitate the strategy that has more resources. For *i* and *j* who have resources $r_{i}$ and $r_{j}$, respectively, the probability of *i* learning from *j* is $p_{ij}$:

(1) $$p_{ij}=\frac{1}{1+e^{\left(r_{i}-r_{j}\right)/\beta }}$$

where $\beta \subseteq [0,1]$ indicates the difficulty of learning, if *i* learns from *j*, *i* copies *j*’s strategy in this round; otherwise, *i* keeps his strategy unchanged. In addition to the learning, the mutation also occurs in each round of the game. The $\left(\alpha \cdot N\right)$ number of individuals in the group mutates where $\alpha \subseteq [0,1]$ is the mutation rate. Each mutated individual changes their strategy to other strategies with equal probability.

In our simulation setup, we choose a group size of N=a·n=100, and they are divided into a=10 subgroups to play the game, each of which has n=10 individuals accordingly. At the beginning of each round of the game, each individual receives m=10 units of resources. Collaborators will contribute u=5 resources, while defectors contribute nothing. The cost to the individual who supports central punishment is $w_{1}=2$, and the total fund required to set up a central agency is w=4. On the other hand, the cost of each peer punishment is $w_{2}=1$. For learning and mutation, β=0.1 and α=0.05. To observe the simulation with different environmental conditions, we repeat the experiments with parameter settings: the intensities of central punishment and peer punishment ranges as $t_{1}\in [2,9.5]$ and $t_{2}\in [1,6.5]$; while the cost required to offset the risk *U* is selected from the set $\left\{15,25,35\right\}$.

## 3. Results

### 3.1. Promotion of Group Collaboration

**Figure 2:**
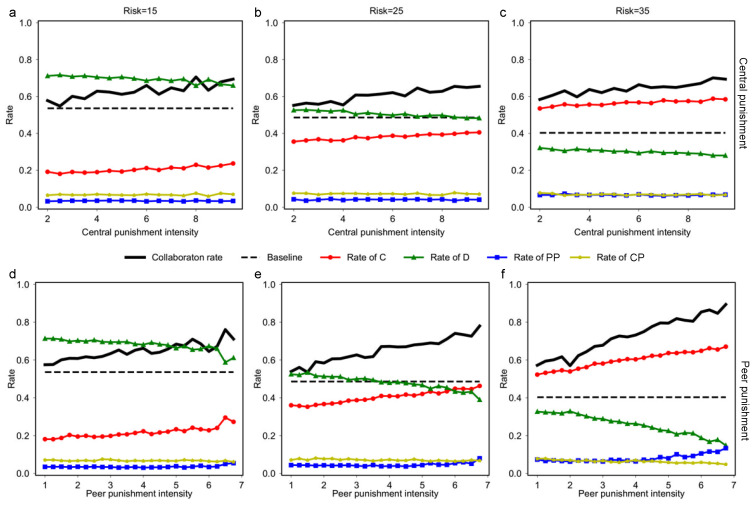
Punishment promotes group collaboration under different risks. Collaboration rate refers to the proportion of people in the population who successfully resist risk through collaboration. (**a**–**c**) in the first row show the change in group collaboration towards $t_{1}$ when $t_{2}=2.5$, and (**d**–**f**) in the second row depict the variation of group collaboration with $t_{2}$ as $t_{1}=5$. The three columns correspond to U=15, 25, and 35, respectively. To make the two punishments comparable, we choose $t_{1}=2\times t_{2}$ to ensure $t_{1}$:$t_{2}$=$w_{1}$:$w_{2}$ so that the ratios of the cost and effect of the two punishment types are the same.

We observe the percentage of people who successfully resist risk through group collaboration (collaboration rate) at different intensities of peer and central punishment. Taking the collaboration rate in the group with only C and D strategies as the baseline, we fix the intensity of peer punishment (in Figure 2a–c) and central punishment (in Figure 2d–f), respectively, to observe the performance of group collaboration when the intensity of the other punishment type varies. As can be seen from Figure 2, the rate of group collaboration in the presence of punishment is always higher than the baseline regardless of the risk itself. In addition, the intensity of punishment positively correlates with the group collaboration rate, which suggests that both central punishment and peer punishment promote group collaboration. Moreover, the result also shows that stronger punishment promotes group collaboration more.

What makes group collaboration successful against risk? We answer this question by analyzing the composition of the population. The conclusions are as follows.

- When comparing the performance of punishment under different risks, we can find that the greater the risk is, the more noticeable enhancement of collaboration by peer punishment with a significantly higher percentage of PP strategies.
- In contrast, the boosting effect of central punishment on the collaboration rate is relatively fixed and does not vary much with different risk levels.
- To summarize, compared with central punishment, peer punishment promotes collaboration more significantly under different risks, and it shows even greater power under high-level risks.

### 3.2. Characteristics of Central Punishment and Peer Punishment

**Figure 3:**
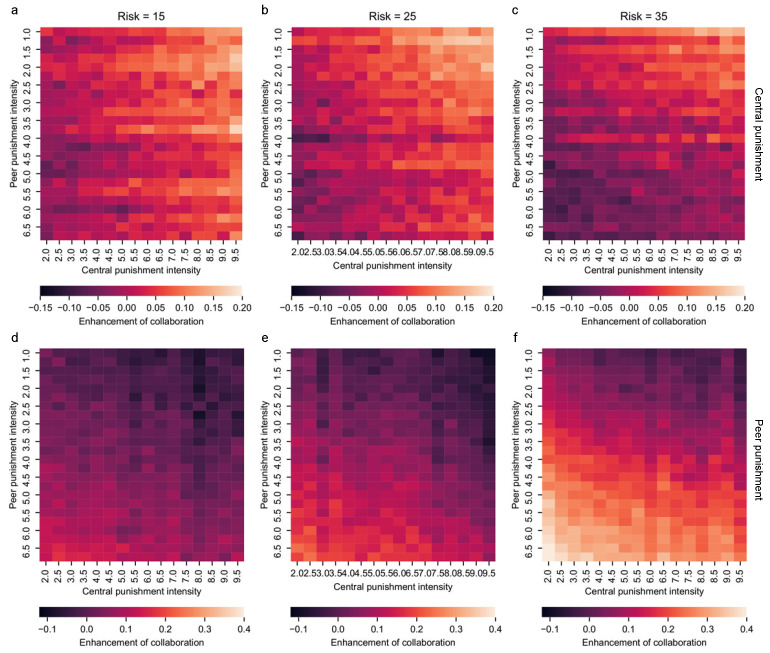
Central punishment and peer punishment’s enhancement of group collaboration. To identify the isolated influence of group collaboration from a single punishment type, we subtract the collaboration rate of one without the targeted punishment method from the one with the four strategies C, D, PP, and CP. The first and second rows demonstrate the enhancement of collaboration by central punishment (**a**–**c**) and peer punishment (**d**–**f**), respectively. The three columns correspond to the punishment performances under different risks U=15 (**a**,**c**), 25 (**b**,**e**), and 35 (**c**,**f**), respectively. Here, $t_{1}$ ranges from 2 to 9.5 and $t_{2}$ ranges from 1 to 6.5 with 0.5 interval. The color represents the enhancement of group collaboration by punishment (see scale), or in other words, the change of collaboration rates.

In Figure 2, by fixing one punishment type’s intensity, we are able to observe the isolated influence of the other punishment type on the collaboration rates. It is important to note that group collaboration is still the result of peer and central punishment joint action. In Figure 3, the isolated observation is conducted by subtracting the collaboration rate with strategies C D and PP (or C D and CP) from the collaboration rate with four strategies of C D PP and CP. The first and second rows in Figure 3 shows the enhancement of collaboration by central punishment and peer punishment, respectively. A common feature of both types of punishment is that increasing the intensity of that punishment always leads to an increase in collaboration, regardless of the risk condition. Specifically, in the first row of Figure 3, as the intensity of the central punishment is increased from 2.0 to 9.5, the colors of Figure 3a–c become lighter. This indicates that the greater the central punishment intensity, the more collaboration is promoted by central punishment. Similar to central punishment, increasing peer punishment intensity from 1.0 to 3.6 also results in lighter colors in both Figure 3d–f, regardless of risk variations.

Central punishment and peer punishment differ in their ability to promote collaboration. As can be seen from the first row in Figure 3, the central punishment promotes collaboration from −0.15 to 0.2; while in the second row, the value varies from −0.1 to 0.4. Negative values imply suppression, and positive values indicate promotion of collaboration. Such value ranges show that central punishment may cause stronger repression of collaboration than peer punishment, and central punishment, especially weak central punishment, suppresses group collaboration. On the other hand, peer punishment has a strong boosting effect on collaboration, which is consistent with the salient effect of peer punishment shown in Figure 2.

Central punishment and peer punishment influence each other in different ways. For central punishment in the first row in Figure 3, changing peer punishment intensity leads to little enhancement of cooperation when risk is 15 (as shown in Figure 3a). While with a risk of 35 (in Figure 3c), the enhancement on collaboration is smaller when peer punishment intensity is bigger than 3.5. As a result, only in the case of high risk is the promotion of collaboration by central punishment influenced by peer punishment. For peer punishment, the promotion of collaboration becomes significantly weaker in Figure 3d–f when central punishment intensity is larger than 7.5. So, the collaboration boosting effect from peer punishment can be suppressed by high-level central punishment, no matter the risk.

How can we balance central punishment and peer punishment to promote group collaboration in real life based on these results? One crucial insight received in this section is that adjusting one punishment type requires consideration of its influence on collaboration and its possible inhibiting effect on the other punishment type. In other words, for either kind of punishment, even though increasing one’s intensity always promotes collaboration, it is at the expense of jeopardizing the other one’s collaboration-boosting ability. Therefore, we need to balance the intensity of both types of punishment. More specifically, when the risk is low, the central punishment heavily limits the enhancement-boosting effect of peer punishment, while peer punishment does not affect central punishment much. So the peer punishment can be fully utilized in such low-risk situations, but the central punishment should avoid high intensity. On the other hand, the two punishment types are strongly mutually constraining when the risk is high. Consequently, neither type of punishment should have a much higher intensity against the other to avoid the constraining. In other words, both punishments need to be used in moderation in such high-risk situations.

### 3.3. Analysis on Cost–Benefit Ratio

**Figure 4:**
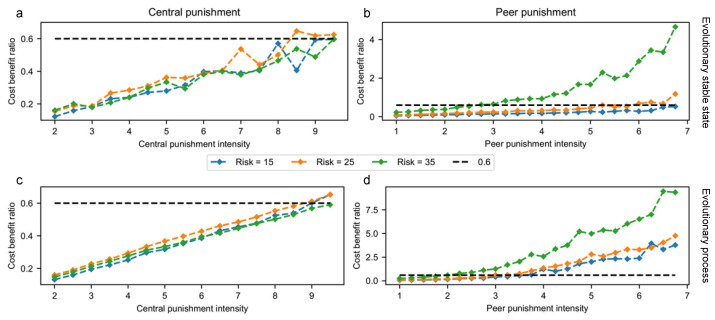
The cost–benefit ratio of central punishment and peer punishment under different risks. The cost–benefit ratio refers to the average resources lost per D individual through central punishment (or peer punishment) to the average cost of punishment from individuals with CP strategy (or PP strategy). The first row corresponds to groups under an evolutionary stable state, which implies the analysis of the last round of evolution. The second row refers to the analysis of the evolutionary process from the beginning to the end. (**a**,**c**) show the cost–benefit ratio of central punishment when $t_{2}=2.5$, while (**b**,**d**) demonstrate the ratio of peer punishment when $t_{1}=5$.

We further explore the cost–benefit ratios of the two types of punishment. As Figure 4a shows, the cost–benefit ratio of central punishment is almost unaffected by risk when the group is in the evolutionary stable state. As the intensity of central punishment increases from 2 to 9.5, the cost–benefit ratio increases from 0.1 to 0.6, showing a positive correlation between the intensity and cost–benefit of central punishment. Similarly, the peer punishment type also shows a positive correlation between its intensity and cost–benefit ratio. In addition, a more noticeable feature of peer punishment is that its cost–benefit ratio increases significantly with the growth of risk level (in Figure 4b). Specifically, with a peer punishment intensity of 6.5, the cost–benefit ratio will explode 7 times from 0.6 to 5 as the risk level grows from U=15 to U=35.

**Figure 5:**
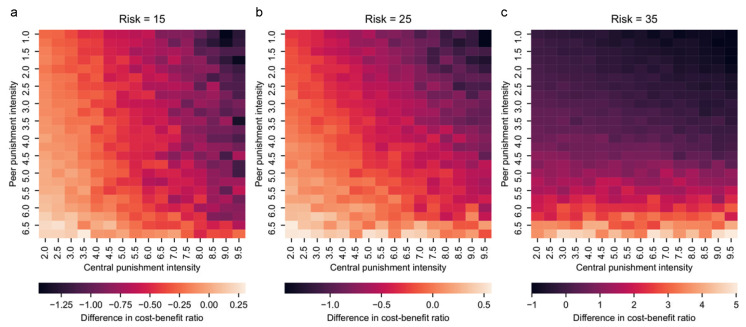
Difference in cost–benefit ratio between peer punishment and central punishment. In a group under central punishment intensity $\hat{t_{1}}$ and peer punishment intensity $\hat{t_{2}}$, the difference in cost–benefit ratio is calculated by deducting the ratio at $t_{1}=\hat{t_{1}}$ from when $t_{2}=\hat{t_{2}}$. (**a**–**c**) correspond to U=15, 25, and 35, respectively. $t_{1}$ is sampled each 1 from 2 to 9.5, and $t_{2}$ ranges from 1 to 6.5 with 0.5 interval. The colors show the difference in cost–benefit ratio (see scale).

Further, we look at the difference in cost–benefit ratio between peer punishment and central punishment to understand the interaction of the two types of punishment as illustrated in Figure 3. In a group under central punishment intensity $\hat{t_{1}}$ and peer punishment intensity $\hat{t_{2}}$, the difference in cost–benefit ratio is calculated by deducting the ratio at $t_{1}=\hat{t_{1}}$ from $t_{2}=\hat{t_{2}}$. As can be seen in Figure 5a, when the risk is 15, increasing the central punishment intensity results in a smaller difference, but the change of peer punishment intensity does not affect the difference. Therefore, it can be concluded that the difference is only affected by the intensity of central punishment. This is consistent with the results in Figure 3c,f that the enhancement from both peer punishment and central punishment are constrained by each other when the risk is high, which is consistent with the results in Figure 3a that the collaboration boosting by central punishment is not constrained by peer punishment. Moreover, such a conclusion explains Figure 3d that the effect of peer punishment is influenced by central punishment under low risk. On the other hand, when risk is 35, the difference in cost–benefit ratio correl.

In addition, we show the cost–benefit ratio during the evolutionary process in Figure 4c,d as a complement to the evolutionary outcome in Figure 4a,b. The cost–benefit ratio of central punishment shows little change between the evolutionary process and the outcome. In contrast, the ratio of peer punishment is significantly higher in the process than in the evolutionary stable state. Its many-to-many relationship nature can explain such characteristics of peer punishment between the punisher and the receiver. Specifically, if there are enough CP individuals, each D individual may receive infinite peer punishment. In comparison, although central punishment requires support from PP individuals, it can only be imposed once on each D individual. Therefore, the cost–benefit ratio of central punishment is almost independent of the share of CP strategy during the evolutionary process, while the cost–benefit ratio of peer punishment varies along with PP strategy rate.

### 3.4. Resource Wastage by the Punishments

**Figure 6:**
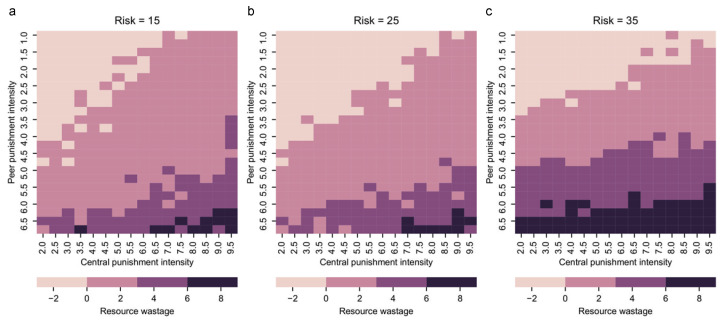
Resource wastage under different peer punishment intensities and central punishment intensities. Resource wastage *k* is calculated as the average of resources contributed by the group members minus the resources needed to cope with the risk. Negative resource wastage means that the group fails to deal with the risk, and everyone loses their resources. (**a**–**c**) denote the resource wastage as U=15, 25, and 35, respectively. Here, $t_{1}$ is sampled each 1 from 2 to 9.5, and $t_{2}$ is sampled each 0.5 from 1 to 6.5. The color indicates the resource wastage (see scale).

The amount of resource wastage is a crucial factor to consider when evaluating group collaboration to counteract risk. In the traditional cooperation model, a higher percentage of group cooperation usually means more benefits for everyone. So promoting a group to achieve full cooperation is the ultimate goal. However, some extra effort will be wasted in the risk-resistant model when the group collaborates more than needed to counteract risk. Thus, a higher collaboration rate is no longer the only target. We have already shown that central punishment and peer punishment can effectively promote group collaboration in the risk-resistant model. In this subsection, we present the resource wastage under different peer and central punishment intensities in Figure 6 to explore the byproduct resource wastage along with collaboration promotions.

The amount of resource waste *k* is defined as the average of resources contributed by the group members minus the resources needed to cope with the risk. Here we divide *k* into four zones to examine waste due to punishments: insufficient contribution zone (k<0), no waste zone (0≤k<3), slight waste zone (3≤k<6), waste zone (k≥6). In a subgroup that fails to be resilient to risk, everyone loses all their resources, which results in a loss of n×m=10×10=100 units of resources. So, a small amount of wasted resources is acceptable compared to the loss of all resources due to unsuccessful risk resistance (k<0). Figure 6 shows that k<6 is the most common case, which is a small amount waste of resources. Thus, we can conclude that punishment promotes collaboration with only a small amount of acceptable resource waste.

Observing the waste of resources due to two types of punishment, we find that resource waste is more strongly influenced by peer punishment than central punishment. Moreover, resource wastage is more likely to occur at high risk and high peer punishment intensity (in Figure 6c). Therefore, in addition to their collaboration promotion influences, resource waste is another essential factor to think about when balancing between central and peer punishment. When the risk is low, even if peer punishment does not affect central punishment’s collaboration promotion, its salient resource waste characteristics should be considered so that we should avoid its high intensity. In high-risk cases, central punishment can be utilized more because of its limited waste of resources.

## 4. Discussion

Although the proposed risk-resistant model is based on the COVID-19 pandemic, it is equally applicable to other scenarios where risks are shared by all, such as global warming, reduced plant and animal diversity, etc. This is because the only characteristic of risk in our model is that it causes losses to all. This feature is not just a description of COVID-19 pandemic. On the other hand, the behavior of people in the model is also generic. People can choose to contribute or not in all cooperation scenarios, and punitive behaviors can be performed directly or indirectly, such as verbal abuse and gossip. Therefore, the model and related findings of this paper can be applied to other risk-sharing scenarios.

In most studies on cooperation, whether based on evolutionary games or behavioral experiments, the payoff matrix of the game is fixed. That is, individuals choose strategies under the condition of a constant cost–benefit ratio of a project [27,31,51]. It usually means a higher level of cooperation between individuals is always preferred. However, more and more realities have far exceeded this constraint, especially the coronavirus epidemic that has pushed a new cooperation scenario of group response to risks to us [53,54]. In such a scenario, the impact of risk is global and mandatory. Specifically, the relationship between the benefits and the costs of the group shows a segmented function. In addition, individuals are not only faced with the option of contribution, but also have to choose how to punish. This requires us to propose a targeted game model to study the law of group evolution and the methods to promote group collaboration. The risk-resistant model in our paper is a new game model for risk response, and more realistic and scenario-specific models are yet to be developed and explored.

As assumed in this study, central punishment and peer punishment do interact to shape the final outcome of group cooperation, but they have different characteristics. Central punishment varies in sensitivity to peer punishment as risk changes, which has far-reaching implications for government management.The effectiveness of central punishment is not affected by peer punishment when the risk is low. Then, the adequate use of peer punishment can further stimulate group performance based on central punishment. In this circumstance, it is not enough for the authority to exercise its power by adjusting its enforcement’s way or strength, which makes autonomy a governing support force that cannot be ignored. For example, in the case of infectious diseases that do not have a high mortality rate, such as the influenza virus, it is more important to cultivate the individual’s sense of self-preparedness based on public health support. However, as the Chinese say, “Water can carry a boat, but it can also overturn it.” When the risk is severe, excessive peer punishment can significantly inhibit the effect of central punishment. Then, the power of peer punishment needs to be used with extra caution. In other words, more focus should be on authority than autonomy. For example, when encountering a significant global risk such as the COVID-19 pandemic, the government needs to play an absolute leading role in organizing the whole society to cope with the risk, rather than leaving the people to choose the way and intensity of input freely. Otherwise, the power of the people could weaken the effect of government policies and even expose the whole society to risks. To achieve equilibrium, authorities need to adapt to the situation and guide the public autonomy to play an appropriate role.

Peer punishment not only has an impact on central punishment, but also has great power of its own. Peer punishment does not involve a lengthy decision-making process and is entirely individual-based, fully mobilizing stakeholders’ enthusiasm. Since peer punishment decentralizes the cost of punishment and superimposes the effect of punishment, public autonomy can achieve a high level of public order at a low cost. So, autonomy is an essential policy option to solve public governance problems. However, it should be noted that although peer punishment has demonstrated great power in the process of group evolution and risky situations, this does not mean that the independent play of peer punishment can inevitably achieve good results. The flexibility of peer punishment is accompanied by uncontrollable consequences when external regulations from the authority is absent. For example, if the punishment target of PP individuals is not only D individuals, i.e., antisocial punishment emerges, then the effectiveness of peer punishment cannot be guaranteed [23,47].

Although peer punishment and central punishment are two distinct manifestations of punishment, they do not exist in opposition. They often interact with and influence each other, explicitly or implicitly, in public governance issues. Peer punishment enhances cooperation through the autonomy of group members. When a significant number of group members hold certain views and attitudes, the authority tend to adjust sanctioning institutions and central punishment based on the will of the people. On the other hand, the long-term effects of central punishment can change group members’ perceptions of fairness and contribution. Different social perceptions accordingly influence the exercise of autonomy, for example, people in different societies have different intensity of peer punishment [55].

The interaction between peer punishment and central punishment is also influenced by other factors outside the model, such as social norms, which we would like to discuss briefly here. Descriptive social norms refer to the existence of a communal perception of a social group in which the majority of the group believes that something can or cannot be done. If the group collectively believes that collaborating against risk is something that should be undertaken, then individuals who behave inconsistently with that perception are more likely to be punished by the group, either by peer punishment or by central punishment. Further, punishment discourages non-collaborative individuals and promotes group collaboration. Thus, descriptive social norms do influence people’s punitive behavior through shared group perceptions and thus influence the contribution of punishment to group collaboration.

## 5. Conclusions

In this paper, we explored the effectiveness of central and peer punishments on group collaboration in risk-resistant contexts. The result shows that both central and peer punishments can promote group collaboration, while peer punishment has a much stronger influence than central punishment. In addition, we analyze the interaction between the two punishment types and find that they affect each other in different ways. Specifically, central punishment constrains peer punishment despite risk levels. Peer punishment, on the other hand, limits central punishment only when the risk level is high. To further understand the interactions between these two punishment types, we analyze and compare the cost–benefit ratio of the two punishments during evolution and when evolution is stable. Last but not least, the resource wastage brought about by the two punishments in facilitating collaboration is discussed. Compared with central punishment, resource waste is more sensitive to the intensity of peer punishment. Considering the trade-off between collaboration effectiveness and waste of resources byproducts, our model suggests peer punishment as the dominant punishment type in a low-risk environment. While at high risk, a better strategy should be to balance the two punishment types, with the central punishment type being slightly more intense. This paper provides a preliminary exploration of central punishment and peer punishment in risk-resistant scenarios, but the interaction and real-world validation of these two remain to be explored. Further research can focus on the mutual shaping of peer punishment and central punishment based on real-world datasets to enlighten the practical application of public autonomy and central governance.

## Data Availability

Data in this paper is available at https://datadryad.org/stash/share/WA2wzcgvT2QhlFkcnWgrvPQPQcwBZ2IOT8TFSQcMahs (accessed on 1 August 2022).
